# From Theory to Practice: Serious Game Education in Singapore and Canada

**DOI:** 10.7759/cureus.99358

**Published:** 2025-12-16

**Authors:** Bill Kapralos, Bina Rai

**Affiliations:** 1 maxSIMhealth Group, Ontario Tech University, Oshawa, CAN; 2 Department of Biomedical Engineering, College of Design and Engineering, National University of Singapore, Singapore, SGP; 3 The N.1 Institute for Health, National University of Singapore, Singapore, SGP

**Keywords:** health professions education, immersive virtual learning environments, instructional design, serious games, virtual simulation

## Abstract

Immersive virtual learning environments (iVLEs) are increasingly used in education, yet their effectiveness is often hindered by educators’ limited knowledge of the design and application of these environments. This chapter thoroughly details two interdisciplinary undergraduate courses whose aim was to introduce students to iVLEs and serious games (SGs) tailored to medical education. One of the courses falls within the Game Development and Interactive Media (GDIM) program at Ontario Tech University in Oshawa, Canada, while the other course falls within the Department of Biomedical Engineering, within the College of Design and Engineering at the National University of Singapore in Singapore, and is available to students within the college. This technical report aims to highlight the importance of the design, development, limitations, and use of SGs while sharing our knowledge and experience through a set of recommendations for those wishing to implement and offer similar courses.

## Introduction

The use of immersive virtual learning environments (iVLEs) within a wide range of educational and training applications has become widespread, given the current generation of learners who are growing up spending a large amount of time playing video games [1,2]. Their use was particularly accelerated during the COVID-19 pandemic, where they shifted from a “backburner training tool to a first-choice strategy for ensuring individual, team, and system readiness”, playing an integral part in facilitating online learning during the abrupt move to remote (virtual) learning given the lockdowns/stay-at-home orders [3]. Although iVLEs include a wide array of digital tools and platforms, such as learning management systems (LMSs) and interactive simulations that utilize extended reality (XR) technologies, including virtual reality (VR) and augmented reality (AR), in this technical report, we are specifically focusing on serious games (SGs). SGs are games created for educational purposes and simulations that may incorporate immersive technologies from the XR spectrum, including AR, mixed reality (MR), and VR. SGs are immersive and interactive in nature, capable of attracting and engaging play-learners for a specific purpose, such as to acquire new knowledge or skills [4], and with respect to students, strong engagement has been associated with higher academic achievement [5,6]. As Katonai et al. [7] outlined, SGs have been linked to various perceptual, cognitive, behavioral, affective, and motivational effects through quick feedback mechanisms, increasing strategic thinking and decision-making, and multiplayer functions offer opportunities for collaborative learning and teamwork development.

Despite the benefits of SGs, their design is a difficult task, and knowledge in game design and development alone is not sufficient to develop effective applications. Many SGs have been dismissed for their poor game design and lack of instructional design (the design features that promote the acquisition of knowledge and skills; see [8]), and often prove inferior to the traditional learning method that the SG was intended to replace [9]. Furthermore, the presence of educational content in a game does not necessarily guarantee its effectiveness. The educational power of any SG depends on a variety of other factors, such as the educational content must be sound, play-learner-appropriate, and presented clearly; the interface must be easily usable by the target audience; and the educational content must be well integrated into the application [10].

The design and development of SGs is an interdisciplinary process, requiring expertise from a variety of fields, including game design, computer science/engineering, education (instructional design), and content expertise (e.g., medicine when considering an SG for medical education). Although designers and developers of SGs are not expected to be experts in instructional design and the specific content area that the game focuses on (e.g., medicine, business, history, etc.), possessing some knowledge in these areas will, at the very least, promote effective communication between the interdisciplinary team members. Further complicating matters is the increasing availability of various authoring platforms that allow educators (who may have limited software development knowledge) to create new or modify existing SGs. One such example is Moirai, a no-code SG authoring platform that allows educators with limited programming experience to use a diagram-based interface to create new modifications and customize SG scenarios focused on decision and communication skills development [11]. Although such tools abstract a large portion of the development details, allowing educators to easily develop SGs tailored to the needs of their students, educators with limited knowledge of SG development, limitations, and their implementation in general can hinder and limit their use as effective teaching tools in the classroom. It is therefore important that educators wishing to incorporate SGs into their classrooms have some knowledge of SGs, including their design, development, playtesting, and limitations.

Recently, several online courses on SGs have become available. For example, The Game Beyond school offers various online courses related to SG design and development, including the SG Design Masterclass course [12], and the Massive Open Online Course (MOOC) List offers the Serious Game Design and Development course [13]. The University of Michigan offers an SG graduate certificate program that requires students to complete three courses either fully online or on campus through hybrid courses. The three courses are i) Foundations of SGs, ii) Theories of Games, and iii) Interaction for Design and Understanding Users. The program provides “game designers, business executives, teachers, and researchers graduate-level insight into SG theories, SG design, and human-centered design” [14]. Despite the availability of these (and other similar courses/programs), the literature reveals little with respect to detailed descriptions of courses regarding SG development. Annetta et al. [15], Chaffin and Barnes [16], and Kapralos et al. [9] are three examples, all of which focus on adult learners at the post-secondary level.

Given the growing popularity of SGs and their benefits [17], along with the importance of knowledge regarding their design and development and their proper use in the classroom, This technical report provides details regarding two courses that focus on SGs, offering a comparative and experiential perspective on SG-focused curricula, highlighting practical insights into course design and implementation and student engagement that can guide educators and researchers both within and beyond game development contexts. Our aim is twofold: i) to highlight the importance of the design, development, and use of SGs, and ii) to share our knowledge and experience with those wishing to implement similar courses so that they can build upon and improve our efforts. It should also be noted that although we are focusing on SGs, many of the concepts we present, including those pertaining to instructional design, are equally applicable to iVLEs and simulations in general [18]. One of the courses (INFR4120) is offered as part of a four-year undergraduate Game Development and Interactive Media (GDIM) degree program at Ontario Tech University in Oshawa, Canada [19] that focuses on the design and development of iVLEs (SGs primarily), with an emphasis on those for medical education. Most students who take INFR4120 are majors within the GDIM program and therefore have a strong technical background, having taken courses in game design/development, 3D modeling and animation, and user interface (UI) design, amongst others. A background in game development is not required for the course, and therefore, the course is open to all students irrespective of their program of study, stressing the interdisciplinary nature of the course. The second course (BN4701, Serious Games for Health) is offered as a technical elective as part of a four-year undergraduate biomedical engineering (BME) degree program within the College of Design and Engineering at the National University of Singapore in Singapore. There are no prerequisites, and a background in game development is not required for the course.

In the following sections, details regarding each course are provided, including an overview of the course, a description of course activities/assignments, and a project. This is followed by a discussion that includes a reflection of course outcomes, comparisons between the two course approaches, recommendations based on our experiences for those wishing to implement/offer similar courses, and a summary.

## Technical report

North American course (INFR4120)

The course INFR4120: Immersive Virtual Learning Environments and Immersive Technologies is an elective course within the GDIM program at Ontario Tech University in Oshawa, Canada. The GDIM program is a four-year degree program that was introduced within the Faculty of Business and Information Technology (FBIT) in 2005 and focuses on the entire development process for video games and interactive media. The program is designed to provide students with a wide range of expertise in game design, development, and interactive media. Each semester, students participate in the Game Development Workshop that integrates knowledge in all disciplines in a back-to-back semester-long team project, allowing students to develop their knowledge and skills in a diverse team environment by designing, developing, extending, and polishing an interactive game that is “delivered” at the end of each school year. Students gain further experiential learning experience through various experiential options such as capstones, internships, and venture creation. First-year courses cover everything from programming to game design to 2D animation, introducing students to basic concepts and ensuring they have the required foundational knowledge. Throughout years two to four of the program, students can choose to focus on the disciplines that interest them the most and gain valuable depth of knowledge in development disciplines such as (but not limited to) game programming, game design, technical art, SGs and simulations, and game user research. Students can acquire business and management knowledge and develop entrepreneurial skills through the core and elective business courses, allowing graduates to quickly advance their careers in the game industry as employees or entrepreneurs in charge of developing and managing their own gaming businesses. Further information regarding the program is available via the program website [19].

INFR4120 was designed and introduced to the GDIM curriculum in 2010, given the growing popularity of SGs, particularly in the fields of medical education, and the lack of availability of courses related to SGs and, more importantly, to their effective design and development. Furthermore, the design and development of SGs and interactive experiences beyond entertainment-based video games is a viable career path for students studying game development. The course is 12 weeks (one semester), and this technical paper will focus on the Fall 2024 offering of the course (September to December 2024), with 51 students, all of whom were from the GDIM program. Although the course covers a wide spectrum of simulation, ranging from physical (e.g., manikin) simulation, virtual simulation, and SGs to various other educational tools such as LMSs, the focus is placed on SGs. In 2014, a fully online version of the course that followed an online problem-based learning approach was designed and offered [9].

The learning outcomes (objectives) of the course are as follows: 1. Understand and discuss the underlying educational theories behind SGs. 2. Understand and discuss the use of SGs within a training and educational setting. 3. Understand how SGs are designed, developed, and integrated into the curriculum. 4. Design and develop effective SGs (that meet their intended objectives). 5. Better evaluate existing SGs used in training and educational settings. 6. Understand and discuss the potential limitations, issues, and open problems associated with SGs.

The course followed a “traditional” delivery approach (e.g., three-hour weekly, in-class lectures) and included a large problem-based learning approach whereby all class activities, including assignments and lectures, were geared towards the group-based final project that saw the design and development of an SG. The course was evaluated along the following: one test (25%), six in-class quizzes/activities (10% total), two assignments (25% total), and a final project (40% total). Greater details regarding each of these components are provided in the following sections.

Course lectures

The course comprised 12 weekly three-hour lectures that included a didactic component (PowerPoint presentation (Microsoft Corporation, Redmond, WA) with many videos and live demonstrations of simulations and SGs, including those from the instructor’s research work in medical education), in addition to class discussion and activities. A summary of the lecture topics is provided below.

Week 1 (Introduction)

Introduction to the course (administrative details, course outline), followed by an introduction to simulation (including a historical overview that emphasized military and medical simulations) and SGs, including a discussion of basic terms/concepts and the motivation for their use.

Weeks 2 and 3 (Instructional Design)

A thorough discussion of the needs and task analysis process emphasizing the importance of thoroughly determining objectives and of consulting with the target audience, subject matter experts, etc., followed by a discussion of various instructional design frameworks (e.g., ADDIE [20]).

Weeks 4 and 5 (Simulations, Game-Based Learning, and Gamification)

A discussion regarding the “simulation spectrum,” including physical simulation, virtual simulation, play, games, video games, SGs, and gamification, and the relationship among them. This also includes an overview of how to design simulations and SGs that create rigorous learning of academic material while appealing to learners.

Week 6 (Learning Theories)

A discussion regarding learning theories (the theoretical basis for SGs and simulation in general), including behaviorism, cognitivism, social constructivism, situated learning, experiential learning, and others. Expertise is also discussed.

Week 7 (Validation and Verification)

A detailed overview of both usability and pre-/post-testing of SGs.

Weeks 8 and 10 (Immersive Technologies and XR)

An overview of XR (AR, VR, and MR in particular) and a discussion of hardware (input/output interaction devices, including head-mounted displays (HMDs), haptics, 3D sound, and camera-based tracking) used to create interactive and engaging SG experiences. An introduction to immersion, perception, and human behavior is also provided.

Week 9 (Mid-term Test)

There are no scheduled lectures.

Week 11 (Ethical Concerns)

An overview of some ethical concerns regarding the use of immersive technologies and SGs, including topics related to health concerns associated with virtual worlds in general (e.g., the prolonged use of head-mounted displays and motion sickness), accessibility, and diversity.

Week 12 (SGs and Immersive Technologies in Medical Education)

A discussion on SGs that incorporate immersive technologies and the design of SGs specifically for medical education.

Students were required to complete a group-based (maximum of five team members) course project that involved the development of an SG for one of two topics specified by the course instructor: i) development of an engaging SG that provides mental health care workers with information they need to understand the safe and appropriate use of psychedelic psychotherapy, an emerging treatment approach that combines the controlled administration of psychedelic substances (such as psilocybin or methylenedioxymethamphetamine (MDMA)) with structured psychotherapy sessions in a clinical setting to treat conditions like post-traumatic stress disorder (PTSD), depression, and anxiety disorders, and ii) development of an engaging SG to teach high school students about the health effects of vaping nicotine. The two topics involved external experts who made themselves available to the students outside of the course lectures. This included an introductory meeting early in the semester where the experts met with students and provided an overview of the topics. Given the limited duration (12 weeks) of the course, emphasis was placed on the educational merit, content, design, justification for the SG, and the design choices students made, and not on the aesthetics (e.g., the “flashy” graphics, animations, etc.). The project included several milestones throughout the semester, which are described below.

Final course project

Topic and Team Selection

Students form teams (on their own) and choose one of the two topics.

Literature Review (Assignment 1; 15%)

The team conducts a literature review of their chosen project topic. Given the limited time frame, the literature was intended to approximate a needs and task analysis.

Project Proposal (Assignment 2; 10%) 

The team expands upon its chosen topics by (i) motivating the area of investigation (explaining why it is interesting), (ii) describing an approach (a “plan of attack”), including what problem analyses, implementation, and testing (if applicable) they intend to perform, and (iii) suggesting possible outcomes.

Project Presentation (10%)

The team prepares a video presentation (and demo) of their project in the form of a YouTube video. Videos are restricted to five minutes in duration. Students are required to provide an introductory section where they introduce the problem and motivate the need to solve the problem (and thus develop their SG), followed by an overview of their SG, including a demo, and concluding remarks. 

Final Report (30%)

The team prepares a thorough and detailed report of their project. The report follows the Institute of Electrical and Electronics Engineers (IEEE) Conference manuscript format and must include i) the learning objectives/outcomes, ii) justification of the design decisions made (with references to prior work), iii) a description of the learning theories employed, iv) a description of the game mechanics employed and why they were employed, and v) a description of each team member’s role.

Course project summary

**Figure 1:**
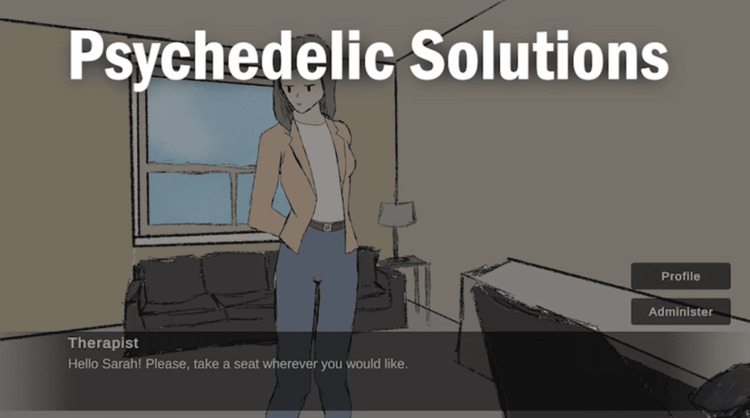
Screenshot of the SG “Psychedelic Solutions: Psychedelic-Assisted Therapeutic Simulator”. The trainee (taking on the role of a medical professional) greets the patient (Sarah, who is shown in the scene) from a first-person perspective. SG: serious game

A total of 14 groups were formed, leading to 14 project submissions, four of which focused on Topic 1 (development of an SG for safe psychedelic psychotherapy training) and 10 of which focused on Topic 2 (development of an SG to teach high school students about the dangers of vaping). Although a complete overview of each of the projects will not be provided here, a brief overview of one project is provided. The project titled “Psychedelic Solutions Psychedelic-Assisted Therapeutic Simulator” was developed by students Willow Forte, Dylan Mills, Vansh Engineer, Sebastian Finkbeiner, and Crystal Polak and targets beginners learning the tools required to perform a psychedelic-assisted therapy session. The game includes a 2D narrative-based play environment that incorporates four main sections: i) patient selection, where the trainee selects one of several patients; ii) preparation sessions, where the trainee works with the patient to choose the correct psychedelic compound to utilize; iii) psychedelic therapy sessions, where the trainee conducts the therapy session, records results, and monitors reactions to the psychedelics; and iv) integration sessions, where the simulated patient and the trainee work to utilize what they learned in their session to apply to the patient’s day-to-day life. The game includes two patient scenarios focusing on six commonly used psychedelics: i) psilocybin, ii) MDMA, iii) ayahuasca, iv) ibogaine, v) mescaline, and vi) ketamine. Each patient includes a profile that includes information such as age, occupation, condition, and background. The trainee is taking on the role of a medical professional. The game includes a dialogue between the patient and the medical professional (trainee) in the medical professional’s office. During the dialogue, the trainee has the option of choosing between a limited number of options, including the option to suggest one of the six psychedelics. At each stage, the trainee is provided with information about their choice and about psychedelics (Figure 1).

Course assignments

The course includes two assignments that are directly related to the course project and thus completed by each team. The first assignment has the teams conduct a literature review on their project topic. Their review must include an introduction section that provides an overview of the subject, the objectives of the literature review, and a description/overview of the process taken to find/obtain their references. For example, teams working on the psychedelic psychotherapy topic might search PubMed and PsycINFO using terms such as "psilocybin-assisted therapy," "MDMA psychotherapy," "psychedelic-assisted treatment," and "mental health worker training," documenting how many papers were found for each combination of search terms and which were selected for inclusion. The review must also include a conclusions section that summarizes their review and provides insight into the open problems in the area and what can be done to improve the area. Although no explicit limit of page length is provided, it is suggested that they aim for 12-15 pages (1.5 line spacing, including references). For the second assignment, each team is responsible for completing a proposal for their chosen project topic, which provides details regarding the approach they will take to implement their SG. For instance, students might identify gaps such as limited training protocols for therapists, a lack of standardized safety screening procedures, or insufficient patient education materials about treatment expectations. Students are reminded that the proposal should convey to the instructor that they possess some knowledge in the area topic and that they have thought about their potential solution. There is no explicit limit on the number of pages, although it is suggested that the proposal be about four pages in length (1.5 line spacing, including references).

Course activities

Students are required to complete six (unannounced) in-class “activities” throughout the duration of the course. The activities are directly related to the material covered during the lectures and the course project and were spread out throughout the semester. The activities generally do not have a correct/incorrect solution, but rather the process of arriving at a particular solution is emphasized. Details regarding each of the activities are provided below:

Activity 1 (Week 1)

Students, working in a group of four or five, assume they are developing an SG on a topic of their choice and are tasked with answering a set of questions regarding the design/development/testing process to the best of their ability without seeking any sources (e.g., the Internet). Although they may not necessarily have the knowledge to answer all the questions, they are asked to do so to the best of their abilities. The purpose of this activity is to i) gauge their knowledge regarding SGs prior to the course, ii) provide them with a glimpse of what may be involved in developing an SG, and iii) provide them with a glimpse of topics that the course will cover. This activity took place during the first lecture.

Activity 2 (Week 3)

Students are tasked with describing the needs analysis process, assuming they are developing an SG for a team of critical care medical professionals who are responsible for stabilizing a patient suffering cardiac arrest. The lecture presented one week before this activity introduces instructional design and provides a brief overview of the needs analysis process. Students are asked to avoid looking up information online and rather answer the question to the best of their ability. The lecture following this activity discusses the needs analysis process in greater detail.

Activity 3 (Lecture 4)

Students are presented with an existing SG called Escape the Experiment, whose purpose is to teach adolescents about the dangers of vaping/e-cigarettes [21], and tasked with accessing/playing the game, providing a review of it, and then suggesting how the game can be improved. There is no particular format for the review, but students are encouraged to consider i) whether they find the SG to be fun and engaging, ii) whether they believe the SG is an effective learning tool, iii) whether they find the SG easy to understand/learn to play, iv) the game’s interface, and vii) whether they believe the SG meets its objectives.

Activity 4 (Lecture 6)

Students are tasked with answering the following questions regarding the Escape the Experiment SG introduced in Activity 3: i) What learning theory(ies) does the game incorporate? ii) Where do you think the game fits on the Dreyfus Expertise scale? iii) Does the game employ implicit and/or explicit learning? iv) What other learning theories could you incorporate to improve the game? and v) discuss how the game can improve player motivation.

Activity 5 (Lecture 7)

With respect to the Escape the Experiment SG introduced in Activity 3, students are tasked with describing the process for determining its effectiveness (e.g., testing to determine whether the SG meets its objectives). This activity took place during the lecture of Week 7 and before any formal discussion regarding effectiveness testing.

Activity 6 (Lecture 12)

Students revisit and answer the questions of Activity 1. Since this activity takes place during the last week of the course (Week 12), all the necessary information to answer the questions was covered in detail throughout the course. Students are also asked to comment on the differences between their responses here and Activity 1. A class discussion follows, where students reflect on differences in their responses to both activities. This activity serves to both review and reinforce the concepts covered throughout the course.

Course evaluations

At Ontario Tech University, all undergraduate courses include anonymous (optional/non-mandatory) student course evaluations that are collected during the last two weeks of the course. Results are made available to the instructors only after final grades have been delivered to the students. The evaluations consist of a series of questions related to the instructor (11 questions), the learning environment (five questions), and the student (five questions). Responses are based on a five-point Likert scale with “1 = Strongly Disagree”, “2 = Disagree”, “3 = Neither Agree nor Disagree”, “4 = Agree”, and “5 = Strongly Agree”. 15 of the 51 students enrolled in the course during the Fall 2024 semester completed the evaluations. All of the responses for each of the 15 students clustered between Agree and Strongly Agree. With respect to the “Instructor” category, the question “The feedback on assignments and/or tests was helpful” resulted in a score of 20% “Agree” and 60% “Strongly Agree”, which highlights the emphasis placed on providing students with feedback. The question “Encouraged student discussion and participation” resulted in a score of 43% “Agree” and 43% “Strongly Agree”, highlighting the emphasis on promoting class discussion. Finally, with respect to the “Student” category, the question “I have achieved the expected learning outcomes of this course” resulted in a score of 53% “Agree” and 33% “Strongly Agree”, providing a good indication that students were overall satisfied with the course.

BN4701 Serious Games for Health (Singapore)

The rationale for implementing a new course focused on designing SGs for biomedical engineering students in Singapore is anchored in the growing importance and application of these games in healthcare settings [22]. SGs have gained significant traction as tools for training healthcare professionals, enhancing patient education, and promoting community health [23, 24]. Hospitals worldwide, including those in Singapore, are increasingly adopting simulation-based training due to its numerous benefits, such as efficient time management, comprehensive competency assessment capabilities, and the ability to simulate diverse medical scenarios [2]. Understanding and mastering the development of SGs tailored to healthcare needs is crucial in meeting the evolving demands of the medical field and will be beneficial for BME students.

Singapore's rapidly aging population presents unique challenges to the healthcare system, including overburdened hospitals and a pressing need for community-based self-care initiatives [25,26]. SGs have emerged as a promising solution, offering applications in the monitoring and enhancement of cognitive function, physical mobility, and rehabilitation. These games not only aid citizens' health maintenance but also alleviate the strain on healthcare facilities by empowering individuals to engage in self-care practices from within their own homes. For BME students, this represents a significant opportunity to contribute to impactful healthcare solutions and to enter a growing niche job market that demands expertise in designing purpose-driven SGs with specific health outcomes.

Current courses in game design and development are readily available, but they typically do not focus on the unique requirements and success indicators of SGs in healthcare [27]. Unlike traditional entertainment games, SGs for health serve distinct purposes, such as improving patient outcomes or enhancing healthcare training, which necessitates a different approach to design and evaluation [28]. This course aims to fill that educational gap, providing students with specialized knowledge and skills to design, develop, and evaluate SGs that meet the specific needs of the health industry. This may enhance their employability and help equip them to make meaningful contributions to healthcare innovation in Singapore and beyond.

Introduction to the course

In the rapidly evolving field of education and training, the integration of SGs can help improve critical thinking, problem-solving, and information retention, and when coupled with AI, it can lead to personalized and optimized educational experiences, providing a revolutionary approach to learning [29]. This course is meticulously crafted to equip higher education students with the necessary interdisciplinary skills to design impactful SGs, focusing on achieving specific learning objectives beyond mere user engagement. At the outset, the course introduces the fundamental concepts of gamification, simulations, and SGs, illustrated through real-world examples from healthcare and community health sectors. These examples serve as a concrete foundation for understanding how games can be leveraged to address real-world challenges in health and wellness.

Safety in aviation has often been compared with safety in healthcare, and as a result, patient safety has often been examined in the context of aviation practice [30]. Forty students first explored examples from the aviation industry and how to apply these to healthcare simulations for enhancing training effectiveness. They then scrutinized the role of gamification as a method for delivering learning content, including when to employ gamification and understanding its two primary types. Effective storytelling in game design was introduced next, focusing on essential components such as characters, plot, setting, and theme. Students were provided with example personas of elderly persons in Singapore that were used to develop their narrative writing skills, with an emphasis on empathy triggers. The use of artificial intelligence (AI) was encouraged to generate images for the personas as well as to draft compelling narratives.

In the first six weeks of BN4701, digital skills were developed through instruction in 2D and 3D game development using the Unity cross-platform game engine, enabling students to create functional game prototypes with basic mechanics, UIs, and interactive elements. Students were guided on how to download and install the Unity Package 6 and Visual Studio. The subsequent component of the course delved into the cognitive neuroscience of memory and learning. Students explored the complex interplay between cellular and molecular storage mechanisms and how the brain supports these processes. This knowledge is crucial, as it underpins the design of SGs that can effectively enhance learning and retention. Students were taught specifically about memory trace consolidation into long-term memory. There was a focus on episodic memory and how emotion is tagged to such memories. With SGs, the possibility of creating episodic memory is very strong. Many 3D immersive games have visual and temporal-spatial relations to provide a strong, rich association between what the player is doing in the event and their long-term memory. The course further explored psychological theories on extrinsic and intrinsic motivation, examining why individuals are drawn to play games. Deci and Ryan’s Self-Determination Theory (SDT) posits that intrinsic motivation is driven by three psychological needs: competence, autonomy, and relatedness [31]. Understanding these motivational drivers and applying self-determination theory is essential for creating SGs that not only educate but also trigger and sustain engagement in players.

Guest lecturers from industry and academia further enrich the curriculum by sharing advanced SG design strategies such as balancing educational content with engagement mechanics, establishing performance metrics, and integrating assessment seamlessly into gameplay. Guest lecturers included Prof. Kevin Yap, an expert in storytelling and AI prompting; Prof. Bill Kapralos, an expert in game and instructional design; and Prof. Brian Stone, an expert in industrial design, UI, and user experience (UX).

**Figure 2:**
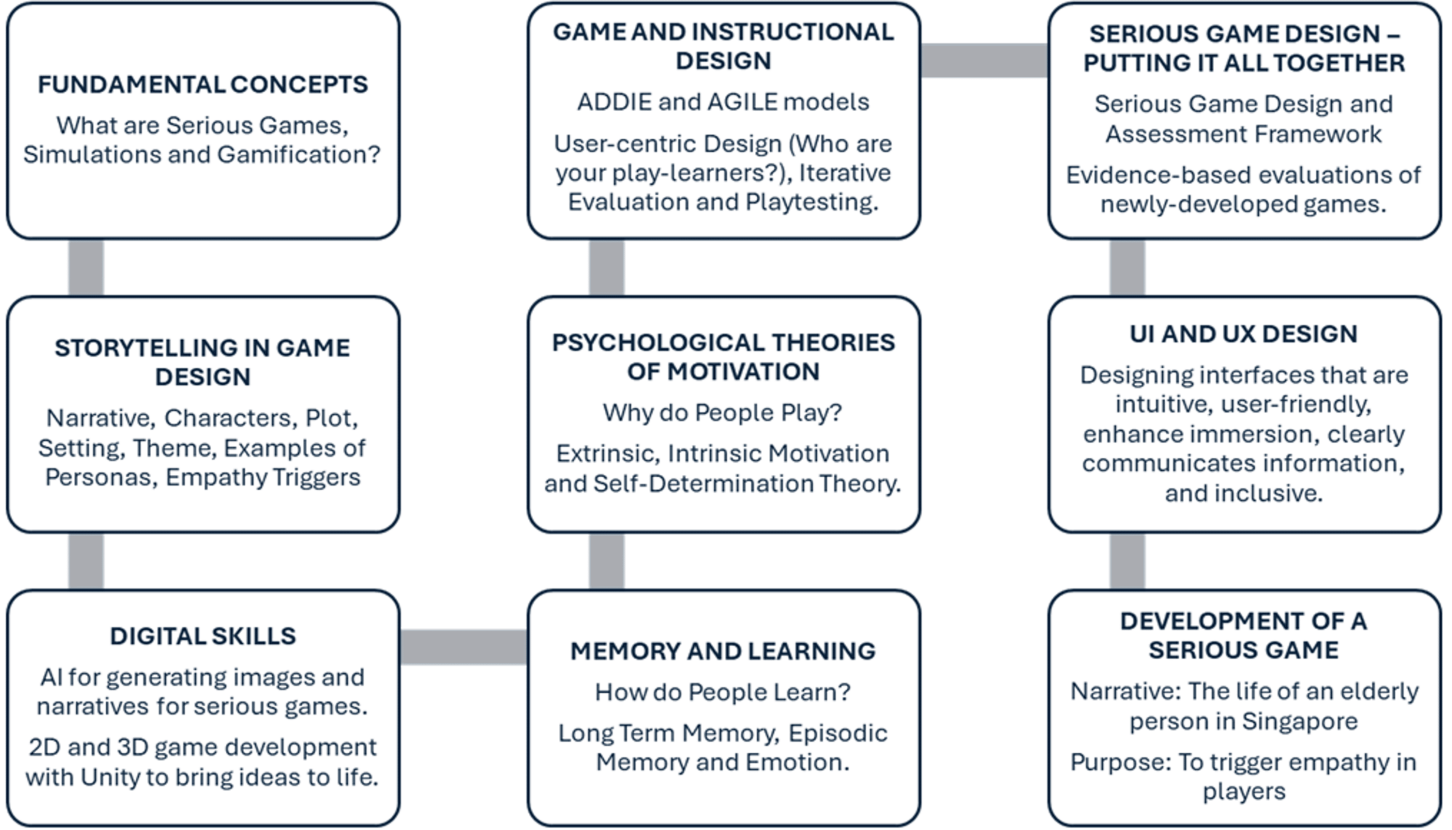
BN4701, Serious Games for Health, course flow diagram; A 13-week interdisciplinary curriculum to learn how to design and develop a SG for healthcare applications.

Students learn about the ADDIE instructional design framework and the Agile software development methodology. The ADDIE instructional design framework includes five phases: Analyze, Design, Develop, Implement, and Evaluate [32], while the Agile methodology suggests an iterative software development process that includes regular short sprint release cycles [33]. A step-by-step approach to SG design is taught, stressing the importance of iterative evaluation and playtesting. An adaptation of the SG Design and Assessment Framework was introduced, which they used to critically evaluate newly developed SGs [26,27]. Finally, students acquire knowledge about UI and UX design principles to create SGs that are intuitive, user-friendly, immersive, and inclusive. By the end of the course, students are expected to proficiently execute an evidence-based approach to designing and developing SGs (Figure 2).

Learning objectives and measurable outcomes

The course is designed with learning objectives to guide students through the intricate process of SG design. Students will learn to recognize the necessity for an instructional design approach that focuses on specific learning objectives. They delve into the cognitive neuroscience underpinning memory and learning, discussing how these biological processes support educational outcomes. Another key objective is to explore psychological learning and motivational theories, particularly how they inform the use of games for effective learning.

The measurable outcomes of this module are structured to assess the students’ grasp of the foundational and advanced concepts introduced throughout the course. Students define SGs and distinguish them from gamification and simulations. They describe the anatomy of the brain and its relationship to memory and learning, and analyze the psychological theories that form the basis of game-based learning. Moreover, students are tasked with applying an adapted version of the Serious Game Design and Assessment Framework [28] to evaluate games designed for health applications, ensuring a robust assessment of their educational value.

Course structure and assessments

The course structure includes a variety of assessments designed to reinforce learning and encourage practical application of the concepts covered. A storytelling session in Week 6 offers a dynamic platform for students to present their narrative ideas, focusing on creativity, clarity, and empathy. This session, contributing 20% to the final grade, fosters collaborative feedback and iterative improvement. A quiz scheduled in Week 7 accounts for 20% of the final grade, assessing students' comprehension of the course material up to that point (see Figure 2 for details regarding the material covered to this point). To further support learning, homework assignments require students to engage in reading responses and game design validation exercises, comprising 20% of the final grade. These assignments are strategically designed to deepen understanding and application of the SG design framework.

The pinnacle of the course is a group project where students, organized in teams, develop a five-minute game using Unity. This project, which accounts for 30% of the final grade, includes workshops to build technical skills, culminating in a pitch and playtest session. Through these diverse learning activities, students emerge as competent designers capable of creating SGs that are not only educational but also transformative in healthcare and community health settings.

Storytelling assignment (individual): crafting empathic narratives for the elderly in Singapore

**Figure 3:**
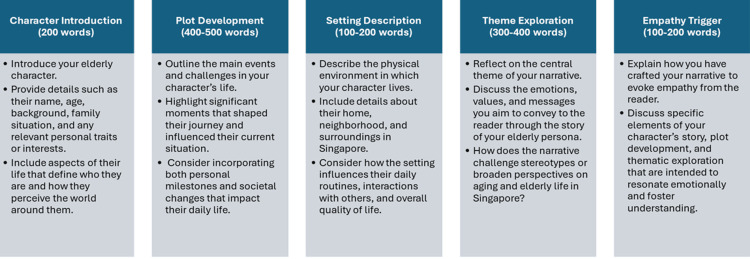
The various components of the narrative for the storytelling assignment.

The objective of the storytelling assignment is to craft a narrative that elicits empathy towards an elderly person living in Singapore. Students are provided with the following instructions: “Imagine you are crafting a narrative that tells the life story of an elderly person residing in Singapore. Your goal is to create a compelling story that evokes empathy and understanding from the reader towards the challenges, joys, and experiences of aging in this cultural context. You are encouraged to be creative while maintaining sensitivity and authenticity in portraying the character and their environment.” Their narrative must include characters, plot, setting, and theme, following the format shown in Figure 3.

Additional guidelines are provided for this storytelling assignment that emphasize the importance of crafting a narrative in a clear and engaging style, suitable for effectively communicating a personal story. The narrative should be between 1,000 and 1,500 words (excluding headings and sketches). Students are permitted to use AI tools such as ChatGPT, Gemini, or Co-Pilot for assistance, but must acknowledge this usage by listing the AI tool used, the prompts and outputs, and how the output is utilized in a table format. Students are allowed to include electronic or hand-drawn sketches to represent key aspects of their character or setting, although this is optional and meant to complement the narrative. Regarding submission requirements, students are requested to submit both their first and second narratives as a typed document. During the Week 6 lesson, students are required to share their first narrative and receive peer and expert (elderly guests) feedback in groups. After making refinements based on this feedback, they submit the feedback, along with the second narrative.

The evaluation criteria for this storytelling assignment are structured to assess various key aspects of the narrative. First, creativity and originality in character development and storytelling are assessed with a total of 8 points awarded for this dimension. This criterion focuses on how uniquely and imaginatively the character and their story are crafted. Second, clarity and coherence of the plot structure and thematic exploration are similarly allocated 8 points. It evaluates how well the story's events and themes are organized and articulated. Third, the narrative's effectiveness in triggering empathy towards the elderly persona is another significant component, with 8 points dedicated to this. This measures how successfully the story engaged the reader's emotions and fostered understanding of the elderly character. Finally, critical reflection based on peer feedback is assessed, with 6 points available for this criterion. This involves evaluating how well the student integrated feedback from peers to refine and enhance their narrative.

Student submissions: storytelling assignment examples

Snippet 1: The Memories of Jennette Tan, Written by Joshua Tan

Character introduction: "I, Marcus Tan Wei Jie, consider myself the luckiest man when I married the love of my life, Jennette. Being alive for 73 years, I do have a lot of memories from my past, which I fondly remember. I remember my childhood seaside house at Haw Par Villa, the art gallery in the National Museum of Singapore, where I first met Jennette, and the west side night markets we would often visit as a couple, as well as the Housing and Development Board (HDB) in Clementi, we moved into after marriage, when the Haw Par Villa house became too old and decrepit to live in."

**Figure 4:**
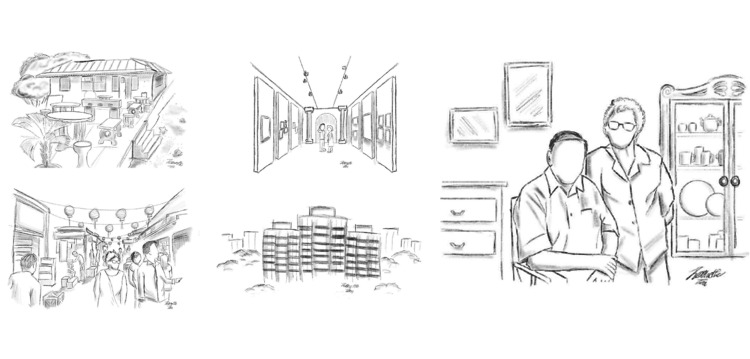
Sample sketches from the student submission

Sample sketches that Jennet drew are shown in Figure 4; in fact, all the artwork in this story was drawn by her. "Jennet always had a creative sparkle in her eyes behind those thick spectacles. Her mind filled with imagination and dreams under those short, curly white hairs. I, as a former contractor, loved observing the beauty in things, and she loved capturing and creating beauty. This kind of pairing was a match made in heaven, and I had looked forward to making more memories with her in our silver years. Making new happy memories, unfortunately, may not be so for Jennette because of one thing... Alzheimer’s disease."

Snippet 2: A Ride Through Memory, Written by Luqman Naqib

Silent in the backseat of his daughter Farah’s car, Pak Rahman listened to the rhythmic wipers and relentless rain. Thunder echoed his heavy heart. Farah chatted about her busy day, her words a soft hum against the raindrops. He gazed out the window at the blurred city lights, a kaleidoscope once sharp and familiar.

He remembered when he was behind the wheel, navigating these streets confidently. As a taxi driver, he'd ferried countless passengers, hands steady, eyes sharp amid traffic. Now, he was a passenger in his own life, reliant on others. The irony wasn't lost on him: the driver reduced to a passenger, the provider now dependent.

“We’re almost there, Ayah,” Farah said, glancing at him. He met her eyes briefly but said nothing. She had explained many times that she was busy with work and his son Amir had moved to Australia. The day care center was the best option. He understood, or told himself he did. Yet understanding didn't ease the sting of feeling like a burden or fill the void of his son's absence.

When they arrived, Farah hurried out, opening the umbrella as she rushed to help him. “Careful, Ayah,” she said, steadying him as he gripped his walker with both hands. The rain made the ground slick, and each step felt precarious, his frail legs struggling to keep up with the support of the walker. Exiting the car had become an ordeal, every movement deliberate and slow. Farah tried to guide him, but he could feel his own weight pulling him down, a stark reminder of his waning strength. “I’ll pick you up after work,” she assured, kissing his cheek. He offered a faint smile, watching her drive away into the rain.

Game development project assignment (group): bringing your narratives to life!

**Figure 5:**
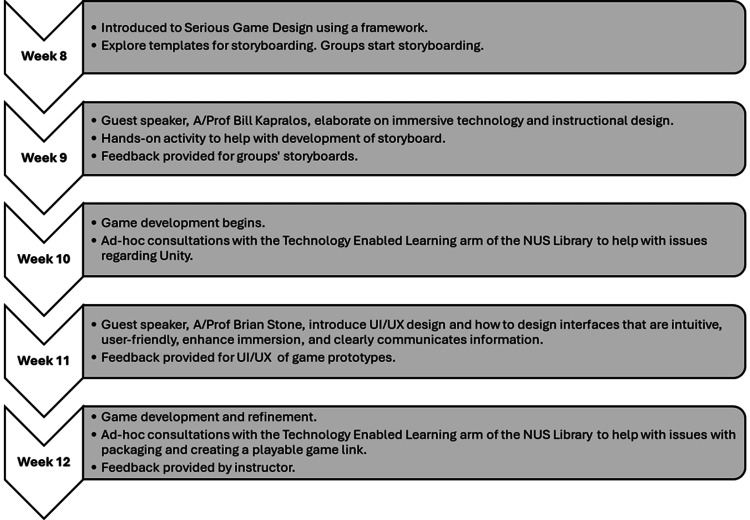
The progressive stages of guided SG development SG: serious game; NUS: National University of Singapore; UI: user interface; UX: user experience

The SG project topic varies for each offering of the course. For this particular course offering, the topic focuses on the elderly in Singapore, given that Singapore is preparing for a super-aged society, and improving societal attitudes towards older persons and aging, which are currently less than ideal [25]. The students are divided into a total of 10 groups with four students each. They are provided a chance to choose their preferred groupings (if any). To foster collaboration and creativity, team members are instructed to connect and exchange their individual narratives from the storytelling assignment. They engage in a discussion to explore each other's stories and select one narrative that resonated with the group. This narrative serves as the foundation for developing an SG with a duration of approximately 10 minutes. The objective is to bring the chosen story to life through gamification. Student groups are free to select any preferred software for game development, such as the Unity game engine, and are provided with a deadline to email the instructor their preferred narrative and software for development. The students then progress into game design and development from Weeks 8-13, as illustrated in Figure 5.

Students introduce their SG as a five-minute pitch at the start of the lesson during Week 13. It was called “Pitch & Play Tuesday”. Five external guests are invited to serve as judges. They consisted of technology and community partners. Once the 10 groups had presented, the playtesting started. There are five game stations set up, and students would move clockwise from one game to the next for an hour before swapping to the next five games for the next hour. Best Pitch and Popular Choice awards are announced at the end of the session. As a final deliverable, student groups submit their storyboard, a playable link with build files, and a recording of the entire gameplay. The rubric used by the judges and instructor for assessment is tabulated in Table 1 and Table 2.

**Table 1:** Rubric for the pitch

No.	Description of criteria	Score (points)
1	Clarity and Coherence	5
	(Pitch is clear and well-organized.)	
	(Pitch is easy to follow.)	
2	Engagement	10
	(Presenter clearly explains the use of game mechanics in the game.)	
	(Game mechanics adopted are engaging and playable.)	
3	Educational Value	10
	(Presenter illustrates how the game effectively triggers empathy towards the elderly.]	
	[Game incorporated instructional design elements such as feedback.)	
4	Innovative Concept	5
	(Pitch was original and creative.)	
	(Innovative application of game elements was presented.)	
	Total Score	30

**Table 2:** Rubric for the game development project

No.	Description of criteria	Score (points)
1	Game narrative adheres to learning objectives in storyboard	20
			2	Game functionality (glitches/bugs) and user interface/user experience	10 + 10
3	General aesthetic qualities (color choice, density, focus of interest, etc.)	5			
			4	Game environment is consistent (assets feel like same materials from the same world)	5
5	Game is engaging and triggers intended emotion	10 + 10			
				Total Score	70

Student submissions: examples of the Game Development Project

Game 1: Life Through a Dementia Lens Developed by Erica Molenberg, Wong Yen Theng, Iman Zulhelmi, and Zhou Christina Lin.

**Figure 6:**
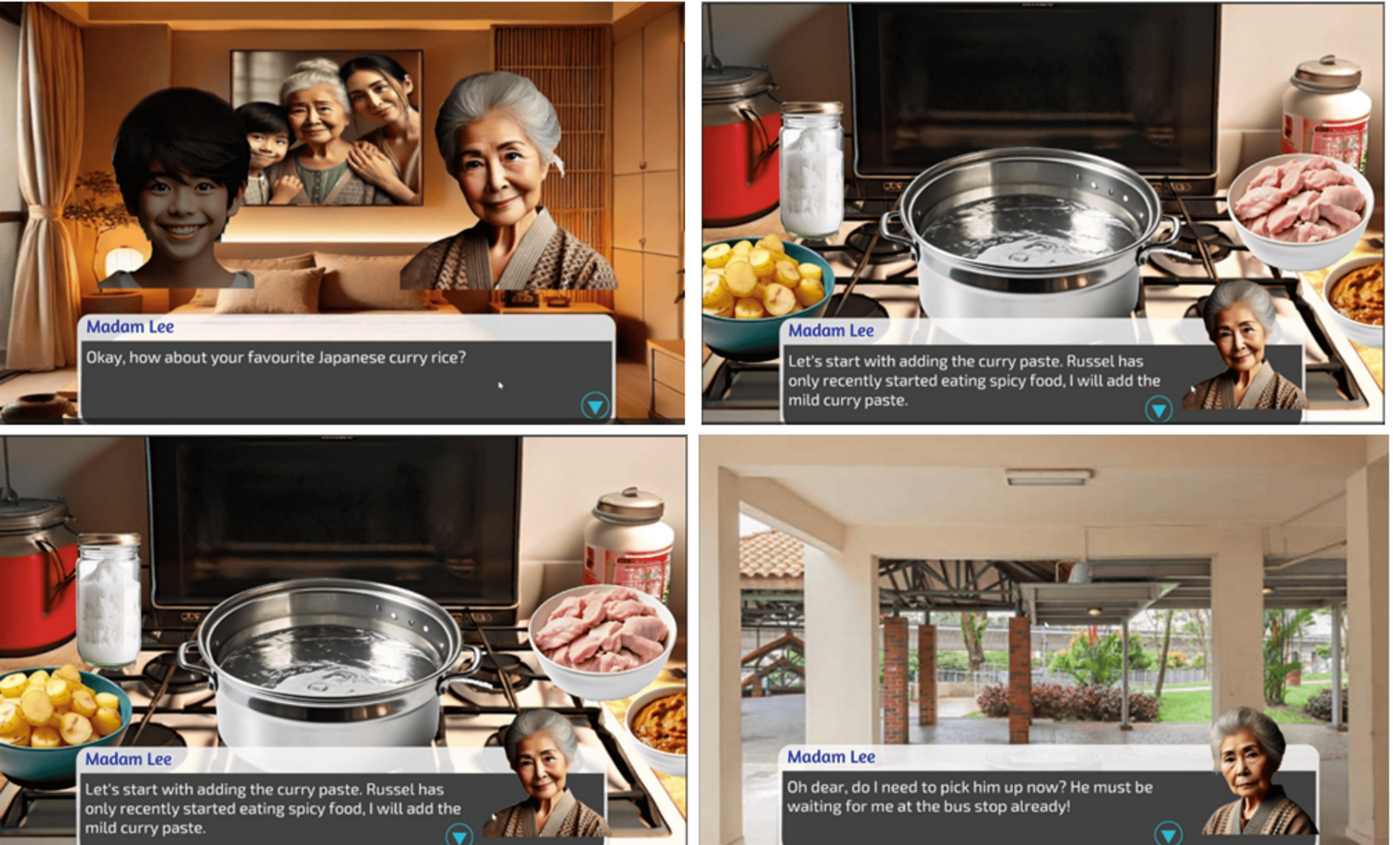
Screenshots from the game, Life Through a Dementia Lens

Description: The game attempts to educate players about dementia by immersing them in the firsthand experiences of Madam Lee, a character with dementia. Players complete various tasks and minigames, such as preparing meals, shopping, and finding their way home, despite memory challenges and distractions. Players navigate through the stages with empathy and patience, understanding Madam Lee's emotional journey and cognitive challenges (Figure 6).

Learning objectives: This game focuses on developing empathy in players by allowing them to experience the challenges faced by patients with dementia, such as memory loss, confusion, and disorientation, from the patients' own perspective. There is an emphasis on the vital role of family and community support, demonstrating how family members, neighbors, and the broader community can significantly assist dementia patients with daily tasks and navigation. Additionally, the aim of the game is to educate players about common dementia behaviors, including memory lapses, difficulties with familiar tasks, and disorientation, to foster a deeper understanding and awareness of the condition.

Game 2: Mom’s Last Bet Developed by Hasina Begum, Park Jun Hyeong, Thiesse Jan Paul Vicco, and Shu Xiaowei

**Figure 7:**
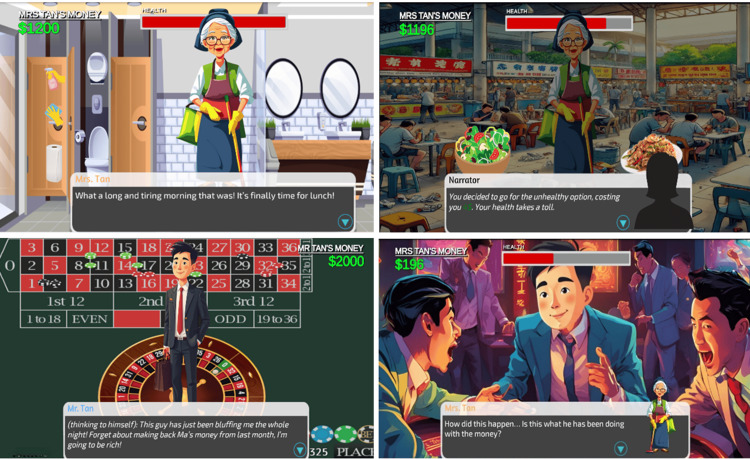
Screenshots from the game, Mom’s Last Bet

Description: Imagine stepping into the shoes of Tan Mei Ling, a cleaner who could be retired but continues to work so her son can afford university and have a better life than she had. Unfortunately, her son drops out, squandering his opportunities on alcohol and dubious gambling. When his mother discovers this, the outcome is uncertain. To find out how the story unfolds, you'll need to play our game (Figure 7). Students designed an innovative game mechanic that allows players to influence their character's health through their decisions, with feedback provided throughout the game.

Learning objectives: This game is designed to bring attention to the unique challenges that elderly parents face as they cope with the realities of aging, while also shedding light on the often-unrecognized sacrifices they make for their children. Players navigate scenarios where their choices are driven by personal judgment, with each decision having a meaningful impact on future game stages. Through this interactive experience, players are invited to gain a richer understanding of the experiences and sacrifices that shape the lives of aging parents.

Evidence of student learning for the Game Development Project

Five external guests were invited to serve as judges for the five-minute pitch delivered by the 10 groups of students. Based on the rubric provided (Table 1), the students were awarded average scores of 4.04 (5) +/- 0.57 for Clarity and Coherence, 7.68 (10) +/- 0.63 for Engagement, 7.84 (10) +/- 0.39 for Educational Value, and 3.68 (5) +/- 0.29 for Innovative Concept. The instructor graded the games based on the rubric shown in Table 2, and average scores of 17.2 (20) +/- 2.62 for “Game narrative adheres to learning objectives in storyboard”, 15.6 (20) +/- 1.07 for “Game functionality and UI/UX”, 3.5 (5) +/- 0.33 for “General aesthetic qualities”, 3.75 (5) +/- 0.54 for “Game environment is consistent”, and 14.3 (20) +/- 1.16 for “Game is engaging and triggers intended emotion” were awarded. The majority of students enrolled in this course are from biomedical engineering rather than computer engineering/science. Given this background and the limited time frame, the development of fully functional games is a noteworthy achievement and a testament to their acquisition of new digital skills. The application of the various interdisciplinary concepts that is required for the design and development of the games is well executed. One component that can be improved is the seamless and timely integration of game elements to evoke the intended emotion in the player, using curated dialogues, audio, and visual triggers.

These quantitative results provide insight into the educational impact of the course. The high scores for engagement and educational value suggest that the emphasis on empathy-driven storytelling and real-world health narratives effectively supported student learning. Meanwhile, moderate UI/UX and functionality scores reflect the students’ developing technical proficiency, which is expected given their biomedical engineering background and limited prior exposure to game engines. The variation in the "intended emotion" metric indicates that integrating emotional cues through dialogue, visual prompts, and audio remains a challenging but essential aspect of SG design.

Student feedback survey for the course

Students were surveyed for their overall opinion of the course and asked to respond on a scale consisting of "Poor", "Very Poor", "Average", "Good", "Very Good". 50% of the respondents rated the course as "Very Good", 43% as "Good", and 7% as "Average". The open-ended feedback from the student perception survey for the course was largely positive, highlighting several key elements that contributed to an engaging learning experience. Students appreciated the inclusion of guest speakers from various fields, which made the lectures informative and attractive. The clear and well-designed flow of the module, along with timely and constructive feedback provided by both the instructor and guests, was also praised. The course content was described as intriguing, with an emphasis on linking lessons back to industry applications, including the use of the Unity game engine and real-world applications such as aviation simulators. The interactive nature of the classes, hands-on activities, a positive classroom atmosphere, and the opportunity to create their own games were common themes in the feedback. Many students found the course content relevant to their interests and appreciated the comprehensive breakdown of topics like gamification, psychology, and game creation.

Some challenges were noted, particularly regarding time constraints for the final project. Students expressed a desire for more time to refine their projects and suggested that the game design process should begin earlier in the course. The difficulty of learning the Unity game engine and developing a game without a background in coding was also mentioned. Some feedback indicated the course felt rushed towards the end, impacting the quality of the final project. Despite these challenges, the course was generally described as fun, engaging, and well-structured, with numerous opportunities for practical application and industry insights.

## Discussion

Here we have presented two undergraduate courses (INFR4120 and BN4701) dedicated to the design, development, and deployment of SGs. INFR4120 is a fourth-year course within the GDIM program at Ontario Tech University and covers the broader spectrum of simulation from physical simulation to virtual simulation and SGs, emphasizing educational frameworks for immersive technologies.

INFR4120 emphasizes understanding and discussing the underlying educational theories behind SGs, including how SGs are designed, developed, and integrated into educational settings. Students are expected to design and develop effective SGs that meet specific educational objectives and evaluate existing SGs in training and educational contexts. Considerable time is also dedicated to discussing the potential limitations, issues, and open problems associated with SGs. BN4701 is a technical elective as part of a four-year undergraduate BME degree program within the College of Design and Engineering at the National University of Singapore in Singapore that targets the application of SGs in healthcare, emphasizing empathy, motivation, and cognitive neuroscience. With BN4701, the learning objectives are centered on designing SGs specifically for health purposes, addressing the unique needs and outcomes of the healthcare industry. The course delves into the cognitive neuroscience of memory and learning and explores psychological theories related to motivation, focusing on how these inform game-based learning in healthcare. A significant objective of the course is to understand and apply motivational theories and empathy-driven game design to create engaging educational tools that trigger and sustain interest in health topics.

Despite these slight differences, both courses emphasize the importance of instructional design. More specifically, students are tasked with recognizing the necessity for instructional design approaches tailored to specific learning objectives in health applications and, in general, ensuring an evidence-based approach to game design. Furthermore, both courses hinge on a “real-world” course project where students can “put into practice” what they learn by designing a SG with real-world objectives, working with medical experts. By completing their projects, students had the opportunity to experience the different stages of SG design and development, and this process was reinforced with the didactic portion in each course, in addition to activities and assignments. In other words, both courses followed a project-based learning approach with a significant experiential learning component. Students received feedback through the entire process from various sources (e.g., peers, instructors, and medical experts), allowing them to refine their work and gain valuable insights. Both courses included detailed rubrics to provide structured feedback on different aspects of the project and course activities/assignments, including educational outcomes, design decisions, user interface, and game functionality. Overall, students in both courses benefit from a combination of instructor, peer, and expert feedback, which helps them refine their projects and develop their skills further.

With INFR4120, students receive guidance and critiques from the instructor and a teaching assistant who evaluate the educational merit, content, and design of their projects with an emphasis on how well the projects meet their intended educational objectives. Throughout the project process, students were encouraged to participate in peer review sessions where they could provide and receive constructive feedback from classmates. Students were also encouraged to test each other’s SG prototypes at various points in the development process (time within several lectures was also allocated for this). This peer interaction helps students view their work from different perspectives and improve upon it. Students also received feedback from the two content experts, who made themselves available to the students throughout the duration of the course. The experts helped the students ensure the accuracy and relevance of the projects. With BN4701, the projects were evaluated by the instructor and external judges during a pitch and playtest session. Feedback focused on clarity, coherence, engagement, educational value, and innovative concepts. During various stages, including storytelling assignments, students also received feedback from peers, which helped them refine their narratives and design elements. Furthermore, through formal feedback sessions, guest speakers and elderly participants provided insights, particularly on narrative empathy and realism, and this served to enhance the authenticity and emotional impact of the projects.

Lessons learned and recommendations

Here we provide an overview of lessons learned after offering these two courses for instructors who wish to implement a similar course. Based on INFR4120, the following recommendations are made.

Start Working on the Project Early

Designing an SG takes a significant amount of time, requiring more time than is available within a one-semester course (12-week duration). It is important for students to form groups and choose a project topic early in the course (ideally during Week 1). Students should also be aware of all requirements and milestones early (also during Week 1). Given the short timeline, emphasis should be placed on a functioning prototype and not a fully polished end product. 

Interdisciplinary Collaboration

Encourage collaboration among students from different disciplines to enhance their learning experience. This approach leverages diverse perspectives and skills, fostering a more comprehensive understanding of complex subjects.

Integration of Theory and Practice

Balance theoretical knowledge with practical application. Ensure that students not only understand educational theories but also know how to apply them in designing functional and effective SGs.

Focus on Educational Objectives

Emphasize the importance of aligning projects with clear educational objectives. This helps students understand the practical implications of their work and ensures the relevance of their projects.

Iterative Feedback

Incorporate regular and timely feedback sessions from instructors, teaching assistants, peers, and external experts to guide students in refining their projects. This iterative process encourages continuous improvement and learning.

With respect to BN4701, the following recommendations are made.

Emphasis on Empathy and Storytelling

Highlight the role of narrative and empathy in game design, particularly in applications such as healthcare and community health. Encourage students to craft stories that resonate emotionally with users, enhancing the impact of their games. Narratives allow us to experience characters' emotions, thoughts, and motivations as if they were our own and stimulate perspective-taking ([34]).

Hands-On Technical Training

Provide students with practical skills in game development tools such as the Unity game engine, ensuring they can effectively bring their ideas to life. Technical workshops can be instrumental in building confidence and competence.

Real-World Relevance

Link course content to real-world applications and challenges. This connection helps students see the practical value of their work and motivates them to engage deeply with the material.

Guest Expertise and Industry Insights

Incorporate guest lectures and industry insights to provide students with current knowledge and real-world applications. Exposure to professionals enriches the curriculum and prepares students for future career opportunities.

More generally, based on our personal experience with both courses, collectively we provide the following advice for instructors who are (or plan on) offering a course on SGs in the future (Table 3). We believe that these tips will help create engaging and effective learning experiences that prepare students for the complexities of designing SGs and immersive technologies.

**Table 3:** Five tips to create a course for teaching serious games design SG: serious game

Tip	Description
Create a supportive learning environment	Foster an environment where students feel supported to experiment, take risks, and learn from failures. This encourages creativity and innovation.
Be adaptive and responsive	Be open to adapting the course structure and content based on student feedback and emerging trends. Flexibility can enhance the learning experience and keep the course relevant.
Encourage reflective practice	Promote reflective practice among students, encouraging them to analyze their learning processes and outcomes. This reflection can lead to a deeper understanding and personal growth.
Real-world relevance	The course should tie to the real-world both with respect to SG examples presented during the lectures and projects. Working on a real-world problem, particularly when this includes external partners (e.g., medical experts), helps to reinforce the importance of SGs and their effective design. Real-world problems can motivate students in general.
External partners	Although we encourage collaboration with external partners particularly with respect to course projects, they should be informed of limitations from the beginning to ensure their expectations are grounded. For example, often (from our experience), medical professionals are often not aware of the intricacies and time commitment required to design and develop an effective SG that is also polished (e.g., high fidelity graphics).

## Conclusions

The design and development of SGs extend beyond game design expertise, requiring an interdisciplinary approach that integrates instructional design, domain-specific knowledge, and game development principles. However, SGs often lack appropriate instructional design, resulting in applications that are inferior to the traditional learning methods that they were intended to replace. Here we have provided a detailed description of two undergraduate courses, INFR4120 and BN4701, offered in Canada and Singapore, respectively, dedicated to the design, development, and deployment of SGs. Both courses emphasized the interdisciplinary and collaborative effort as well as the importance of instructional design to develop effective applications. Students found both courses engaging, enjoyable, and fun, and through experiential learning (course project), gained practical experience in designing, developing, and deploying SGs, while appreciating the complexities involved. Based on student feedback, most students also believed that they met the expected course objectives, and this was validated by the completion of their course projects. We provided a set of recommendations that we believe will help readers interested in offering similar courses.
